# Direct ABCA7 high‐risk allele modulates medial temporal lobe dynamic network flexibility among cognitively healthy older African Americans

**DOI:** 10.1002/alz.095316

**Published:** 2025-01-09

**Authors:** Alyssa Arnasalam, Mustafa Sheikh, Miray Budak, Wiktoria Piaszczynska, Arcande Oyono, José Mojica Perez, Bernadette A. Fausto, Mark A. Gluck

**Affiliations:** ^1^ Rutgers University–Newark, Newark, NJ USA

## Abstract

**Background:**

The medial temporal lobe (MTL) is the earliest region to display atrophy in Alzheimer’s disease (AD)^1^. *Apolipoprotein E (APOE‐ε4)* and *ABCA7‐80* (rs115550680), two genes involved in lipid metabolism, are the strongest heritable contributors to AD in African Americans^2^. However, the longitudinal influence of these genes on MTL dynamic network flexibility, a novel neuroimaging marker of preclinical AD, is unknown. This study investigated the influence of *APOE‐ε4* and *ABCA7‐80* high‐risk alleles on MTL dynamic network flexibility longitudinally among cognitively healthy older African Americans.

**Method:**

135 participants (Age≥60, Mean_baseline age_ = 68.07, SD_baseline age_ = 7.31, Mean_education_ = 13.56, SD_education_ = 2.99) from the longitudinal study, *Pathways to Healthy Aging in African Americans* conducted at Rutgers University–Newark, completed saliva collection for genotyping and ≥1 neuroimaging visits across 4 years. Associations between *APOE‐*ε4 and *ABCA7‐80* on MTL dynamic network flexibility across time were assessed using linear mixed models.

**Result:**

At baseline, the *APOE‐ε4* allele had no significant relationship with MTL Dynamic Network Flexibility (*ß = 0.034, p = 0.297*), whereas *ABCA7‐80* was significantly associated with MTL Dynamic Network Flexibility (*ß = ‐0.124, p = 0.003*). The *ABCA7‐80* x time interaction was significantly associated with MTL Dynamic Network Flexibility (*ß = ‐0.074, p = 0.029*), while there was no such significant *ApoE‐ε4* x time interaction (ß_ApoE*Instance_ = ‐0.017, p_ApoE*Instance_ = 0.53).

**Conclusion:**

There were baseline and longitudinal differences on MTL dynamic network flexibility between *ABCA7‐80* but not *APOE‐ε4* risk groups among cognitively healthy older African Americans. *ABCA7‐80* may have greater influence than *APOE‐ε4* in the early MTL network changes associated with AD risk in older African Americans.

